# Short-term head-down bed rest microgravity simulation alters salivary microbiome in young healthy men

**DOI:** 10.3389/fmicb.2022.1056637

**Published:** 2022-11-10

**Authors:** Hui Sun, Qian Zhou, Pengyan Qiao, Di Zhu, Bingmu Xin, Bin Wu, Chuhua Tang

**Affiliations:** ^1^306th Clinical College of PLA, The Fifth Clinical College, Anhui Medical University, Beijing, China; ^2^Department of Stomatology, PLA Strategic Support Force Characteristic Medical Center, Beijing, China; ^3^Engineering Research Center of Human Circadian Rhythm and Sleep (Shenzhen), Space Science and Technology Institute (Shenzhen), Shenzhen, China; ^4^China Astronaut Research and Training Center, Beijing, China

**Keywords:** head-down bed rest, microgravity, saliva, microbiome, 16S rRNA sequencing

## Abstract

Microgravity influences are prevalent during orbital flight and can adversely affect astronaut physiology. Notably, it may affect the physicochemical properties of saliva and the salivary microbial community. Therefore, this study simulates microgravity in space using a ground-based −6° head-down bed rest (HDBR) test to observe the effects of microgravity on oral salivary secretion function and the salivary microbiome. Sixteen healthy young male volunteers were recruited for the 15-day −6° HDBR test. Non-stimulated whole saliva was collected on day 1 (pre-HDBR), on days 5, 10, and 15 of HDBR, and day 6 of recovery. Salivary pH and salivary flow rate were measured, and the V3–V4 region of the 16S rRNA gene was sequenced and analyzed in 80 saliva samples. The results showed that there were no significant differences in salivary pH, salivary flow rate, and alpha diversity between any two time points. However, beta diversity analysis revealed significant differences between pre-HDBR and the other four time points. After HDBR, the relative abundances of *Actinomyces*, *Parvimonas*, *Peptostreptococcus*, *Porphyromonas*, *Oribacterium*, and *Capnocytophaga* increased significantly, whereas the relative abundances of *Neisseria* and *Haemophilus* decreased significantly. However, the relative abundances of *Oribacterium* and *Capnocytophaga* did not recover to the pre-HDBR level on day 6 of recovery. Network analysis revealed that the number of relationships between genera decreased, and the positive and negative correlations between genera changed in a complex manner after HDBR and did not reach their original levels on day 6 of recovery. PICRUSt analysis demonstrated that some gene functions of the salivary microbiome also changed after HDBR and remained significantly different from those before HDBR on day 6 of recovery. Collectively, 15 days of −6° HDBR had minimal effect on salivary secretion function but resulted in significant changes in the salivary microbiome, mainly manifested as an increase in oral disease-related bacteria and a decrease in oral health-related commensal bacteria. Further research is required to confirm these oral microbial changes and explore the underlying pathological mechanisms to determine the long-term effects on astronauts embarking on long-duration voyages to outer space.

## Introduction

During the last 60 years of crewed space flight, several hazards of microgravity to human physiology have been identified, such as muscle atrophy, bone loss, and immune system dysregulation (Blaber et al., 2010). Dental emergencies have been reported at Salyut 6 and Mir space stations (Ball and Evans, 2001; Barratt and Pool, 2008). However, no in-flight dental emergencies were reported during the Apollo program in the United States (Johnston et al., 1975). Five of the 33 Apollo astronauts required dental treatment 90 days before the flight (Johnston et al., 1975). Among these, one pre-and one post-flight case of pulpitis were observed. Its occurrence during space flight could have prevented the astronauts from completing their mission.

The human oral cavity is a complex micro-ecosystem and is the second largest reservoir of bacteria in the body, containing over 700 species of bacteria (Deo and Deshmukh, 2019). To the best of our knowledge, oral microorganisms are closely associated with oral diseases such as caries (Dinis et al., 2022), periodontitis (Plachokova et al., 2021), and oral cancer (Zhang et al., 2019); they are also involved in various systemic diseases such as diabetes (Matsha et al., 2020), Alzheimer’s disease (Dominy et al., 2019), pancreatic cancer (Fan et al., 2018), and colorectal cancer (Wang et al., 2021). Previous and recent studies have also suggested that oral soft and hard tissues, including salivary glands (Groza et al., 1981, 1983; Mednieks and Hand, 1987; Mednieks et al., 2014; Dagdeviren et al., 2018a; Hand et al., 2020), mandibles (Simmons et al., 1983; Ghosh et al., 2016; Dagdeviren et al., 2018b), and teeth (Rosenberg et al., 1984; Dagdeviren et al., 2018b), are affected by spaceflight. The risk of dental diseases, such as caries and periodontitis, increases in a microgravity environment (Rai and Kaur, 2011). During the Skylab space station mission, an increase in gingival inflammation, specific anaerobic and cariogenic streptococci, and secretory immunoglobulin A was observed in the saliva of in-flight astronauts (Brown et al., 1974, 1976, 1977). Cheng et al. (2014) reported that isolates of *Streptococcus mutans* cultivated in simulated microgravity environments demonstrated increased acid tolerance, altered biofilm structure and extracellular polysaccharide distribution, and an enhanced ability to compete with *Streptococcus sanguinis*, resulting in changes in caries-causing properties. Zhou et al. (2015) revealed that a short-term simulated weightless environment affects the growth and ability of *S. mutans* to synthesize extracellular polysaccharides. Orsini et al. (2017) reported that the metabolism, physiology, and gene expression of *S. mutans* were altered in simulated microgravity environments, which affected cell aggregation and antioxidant stress resistance. Rai et al. (2011) observed that after 60 days of head-down bed rest (HDBR) testing, individuals exhibited elevated markers of oxidative stress in their saliva and a trend toward increased probing depth and attachment loss in the periodontium. Therefore, the effects of microgravity on oral microbes may pose greater challenges to the oral health of astronauts with increased spaceflight duration.

Saliva is a crucial component of the internal environment, and changes in its effective components, pH, and flow rate are closely related to caries and periodontitis (Gao et al., 2016; Lăzureanu et al., 2021). Saliva collection is simple, safe, and noninvasive (Kaczor-Urbanowicz et al., 2017). It also aggregates bacteria and metabolites from other oral ecological niches and represents the entire oral microbiome (Yamashita and Takeshita, 2017). Therefore, saliva is probably the most widely used sample source for oral microbiological investigations. Many studies have suggested that microgravity can alter microbial processes, including growth, morphology, gene expression, virulence, and metabolism (Wilson et al., 2007; Vukanti et al., 2012; Sharma and Curtis, 2022). However, only a few studies have been conducted on the effects of space flight on oral microbial populations (Avila-Herrera et al., 2020; Urbaniak et al., 2020; Morrison et al., 2021), and the effects of microgravity on the composition and function of these microbial communities are poorly understood.

With the rapid development of culture-independent molecular biology techniques, 16S rRNA gene sequencing technology has facilitated efficient bacterial identification (Johnson et al., 2019). Therefore, we monitored the pH and flow rate of saliva, and 16S rRNA gene sequencing technology was performed to observe the effects of microgravity on oral salivary secretion and salivary microbial communities through a 15-day −6° HDBR experiment. We believe that the findings of our study would provide a theoretical basis for the prevention and treatment of oral diseases in astronauts.

## Materials and methods

### Participants

The recruitment criteria for this study were as follows: (1) male, 18–45 years old, 160–175 cm in height, 50–80 kg in weight, and healthy; (2) no oral infectious diseases; (3) non-smoking and not drug-dependent; (4) no systemic diseases; and (5) no history of mental or psychological illness. After medical examination and selection, 16 healthy men were recruited. None of the participants withdrew during the experiment. The bed rest protocol complied with the ethical standards of the Declaration of Helsinki and was approved by the Ethics Committee of Space Science and Technology Institute (Shenzhen; approval number: SISCJK202009001). All participants provided written informed consent.

### Head-down bed rest test

The head-down bed rest test, in which healthy participants are confined to bed in a 6° head-down tilt position, is a well-established model for some of the adaptations experienced by astronauts during spaceflight, and on occasion is used as a pre-flight tool to collect experimental data in crews (Fortney et al., 1996; Pavy-Le Traon et al., 2007; Hargens and Vico, 2016; Pandiarajan and Hargens, 2020). The study period was 28 days, which included a seven-day adaptation period, a 15-day HDBR period, and a six-day recovery period. There were two participants in each room. All eight rooms were kept at a temperature of about 25°C and were equipped with cameras to record daily activities. The following controls were performed to strictly control the −6° head-down bed condition for a more accurate microgravity simulation: (1) Before the experiment, all individuals were coached in oral hygiene by a professional dentist to ensure that the method and time of tooth brushing were consistent. (2) A common brand of toothbrush and toothpaste was used throughout the experiment. (3) The daily schedule of the participants was consistent and included waking up at 7:00 a.m. and sleeping at 10:30 p.m. (4) A uniform diet was provided daily by a dietitian to ensure standard nutrient intake levels. (5) Participants were confined to bed 24 h per day, and all activities such as bathing, eating, urinating, and defecating were performed in bed. Participants were allowed to turn over and onto their sides but were not allowed to raise their heads.

### Saliva sample collection

Non-stimulated whole saliva samples were obtained at five time points during the study: day 1 before HDBR (AS group), day 5 of HDBR (BS group), day 10 of HDBR (CS group), day 15 of HDBR (DS group), and day 6 of recovery (ES group). Saliva samples were collected at 9:00 a.m. each time, and the sampling time was approximately 20 min. The participants fasted for 1 h and were asked to gargle with 30 ml of deionized water before sample collection. Non-stimulated whole saliva was collected for 3 min with a 5 sterile ml saliva DNA collector (tube A) for bioinformatic analysis. And 3 ml saliva was collected in a 5 ml Eppendorf tube (tube B) in the same way for pH evaluation.

### Measurement of salivary secretion function

The tube A was weighed before and after collection using a model ME55 electronic analytical balance (Mettler Toledo, Columbus, OH, United States). The difference between before and after weighing was the saliva weight. The salivary flow rate was measured in grams per minute (g/min). After weighing, a saliva preservation solution (Charm Biotech, Wuhan, China) was added to tube A and mixed evenly before being immediately stored in the −80°C refrigerator until further use. Meanwhile, the pH of the saliva in tube B was measured using a pH meter (ThermoFisher, Waltham, MA, United States).

### Saliva sequencing process

Bacterial genomic DNA was extracted from the samples using the cetyltrimethylammonium bromide (CTAB) method (Zhang and Wang, 2017). The purity and concentration of DNA were detected using 1% agarose gel electrophoresis. Appropriate samples were placed in centrifuge tubes and diluted to 1 ng/μl with sterile water. The bacterial 16S rRNA universal primers 341F (5′-CCTAYGGGRBGCASCAG-3′) and 806R (5′-GGACTACNNGGGTATCTAAT-3′) were used for PCR amplification of the V3–V4 region. The PCR mixture was composed of 25 μl of Phusion® High-Fidelity PCR Master Mix (New England Biolabs, Ipswich, MA, United States), 1 μl of each forward and reverse primer, 2.5 μl of DNA template, and 8 μl of ultra-pure water, amounting to a total volume of 37.5 μl. The PCR amplification conditions were set as follows: initial denaturation at 98°C for 1 min, followed by 30 cycles of denaturation at 98°C for 10 s, annealing at 50°C for 30 s, primer extension at 72°C for 30 s, and a final extension step at 72°C for 5 min. The PCR products were analyzed directly on a 2% agarose gel. Then, PCR products were mixed in equidensity ratios, and samples with a clearly defined band at the expected 500 bp were purified using a GeneJET Gel Extraction Kit (Qiagen, Hilden, Germany). A TruSeq® DNA PCR-Free Sample Preparation Kit (Illumina, San Diego, CA, United States) was used for library construction, following the manufacturer’s recommendations. The constructed library was quantified using a Qubit fluorometer (ThermoFisher, Carlsbad, CA, United States) and Agilent Bioanalyzer 2,100 system. Finally, the library was processed for sequencing on a NovaSeq platform (Illumina, San Diego, CA, United States) at Beijing Novogene Technology Co., Ltd. (Beijing, China).

### Bioinformatic and statistical analyses

After truncating the barcodes and primer sequences, the raw tags were obtained by splicing the paired-end reads corresponding to each sample using FLASH (v1.2.7; Magoc and Salzberg, 2011). In accordance with the QIIME (v1.9.1; Caporaso et al., 2010) quality control process, clean tags were obtained after filtering low-quality and short-base-length tags. The tags were then compared with those in the Silva database^1^ (Glöckner et al., 2017), and chimera sequences were removed. Finally, the effective tags were obtained. The effective tags were clustered into operational taxonomic units (OTUs) at a similarity level of 97% using Uparse software (v7. 01001). Then, the Silva database was used to annotate all representative sequences of OTUs into different biological information at each taxonomic level based on the Mothur algorithm. The Simpson, Observed species, Chao1, and phylogenetic diversity (PD) whole-tree indices were calculated to estimate alpha diversity. Principal coordinates analysis (PCoA; Lozupone et al., 2007) based on weighted UniFrac distance and non-metric multidimensional scaling (NMDS; Kruskal, 1964) based on Bray–Curtis distance was performed to assess beta diversity. PCoA and NMDS plots were generated using R software (v2.15.3). Non-parametric permutational multivariate analysis of variance (ADONIS; Anderson, 2001) and analysis of similarities (ANOSIM; Chapman and Underwood, 1999) were used to test the significance of differences in community structure between groups. The Shapiro–Wilk test was used to assess data distribution. To compare the differences in salivary pH, flow rate, alpha diversity, and taxa at all levels, repeated-measures one-way analysis of variance (ANOVA) followed by Tukey’s multiple comparisons test (normal distribution) or Friedman test with Dunn’s multiple comparisons test (non-normal distribution) was performed using GraphPad Prism (v8.0.1). Spearman’s correlation coefficient was used to construct the co-occurrence of the salivary microbiome. Genera with a mean relative abundance of <0.005% in each group were removed. The networks of the five groups with an |R| value >0.6 and deemed significant (*p* < 0.05) were graphed using Graphviz (v2.38.0). The metabolic function between groups was analyzed using a paired Student’s t-test with statistical significance set at *p* < 0.05.

## Results

### Demographic data

Demographic data are presented in Table 1. Sixteen healthy young men with an average age of (28.94 ± 3.40) years were enrolled in this study. There were no statistically significant differences in salivary pH and flow rate between any two groups (*p* > 0.05). However, compared with the AS group, salivary pH tended to decrease, and salivary flow rate tended to increase in the BS, CS, and DS groups. All returned to pre-HDBR levels on day 6 of recovery.

**Table 1:** Demographic data of the five groups.

	AS	BS	CS	DS	ES
Age (years)	28.94 ± 3.40				
Salivary pH	7.17 ± 0.33^a^	7.08 ± 0.29^a^	7.10 ± 0.24^a^	7.06 ± 0.26^a^	7.31 ± 0.36^a^
Salivary flow rate	0.55(0.43,0.72)^b^	0.61(0.44,0.82)^b^	0.58(0.36,0.90)^b^	0.60(0.42,0.73)^b^	0.49(0.34,0.77)^b^

Values are presented as mean ± standard deviations or median values (interquartile range). AS, day 1 before HDBR; BS, day 5 of HDBR; CS, day 10 of HDBR; DS, day 15 of HDBR; ES, day 6 of recovery.

a *p* > 0.05 by repeated-measures one-way ANOVA between groups.

b *p* > 0.05 by Friedman test between groups.

### Quality of The sequencing data

After sequence assembly, data filtration, and chimera removal, 4,451,246 high-quality sequences from 80 saliva samples were obtained, with an average number of 55,641 sequences per sample. The data pre-processing statistics and quality control data are shown in Supplementary Table 1. All sequences with ≥97% similarity were clustered into 5,216 OTUs, including 3,544 (67.94%) OTUs annotated in the database, and 1,345 OTUs annotated on the genus level. We identified 25 phyla, 36 classes, 75 orders, 137 families, 298 genera, and 381 species in the salivary microbiome.

### Bacterial diversity and community structure

**Figure 1:**
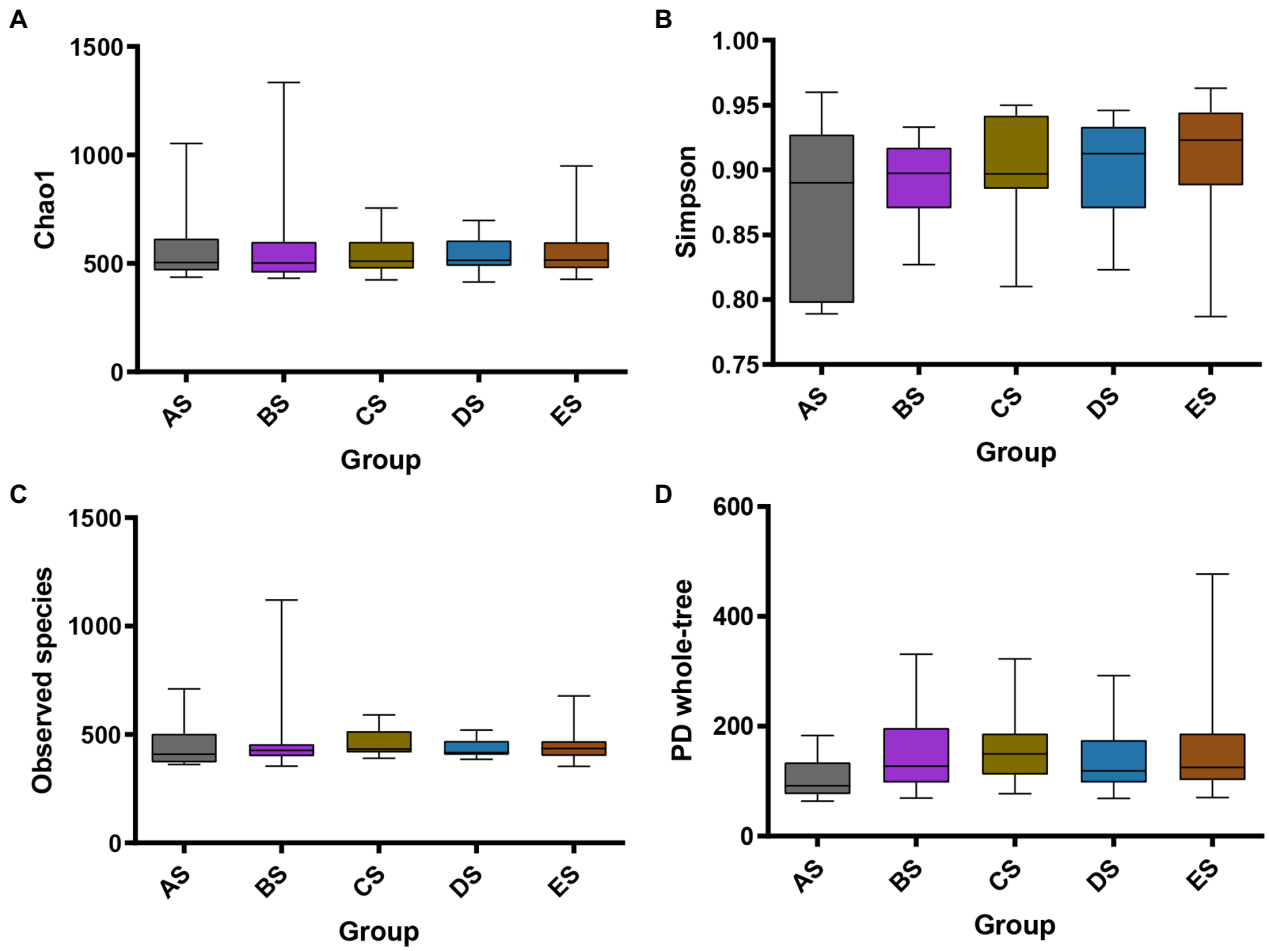
Alpha diversity of saliva samples among the five groups. Box plots depict bacterial diversity according to the Chao1 **(A)**, Simpson **(B)**, Observed species **(C)**, and PD whole-tree **(D)** indices. Statistical analyses were performed using the Friedman test with Dunn’s post-hoc test. There was no statistically significant difference between any two groups.

**Figure 2:**
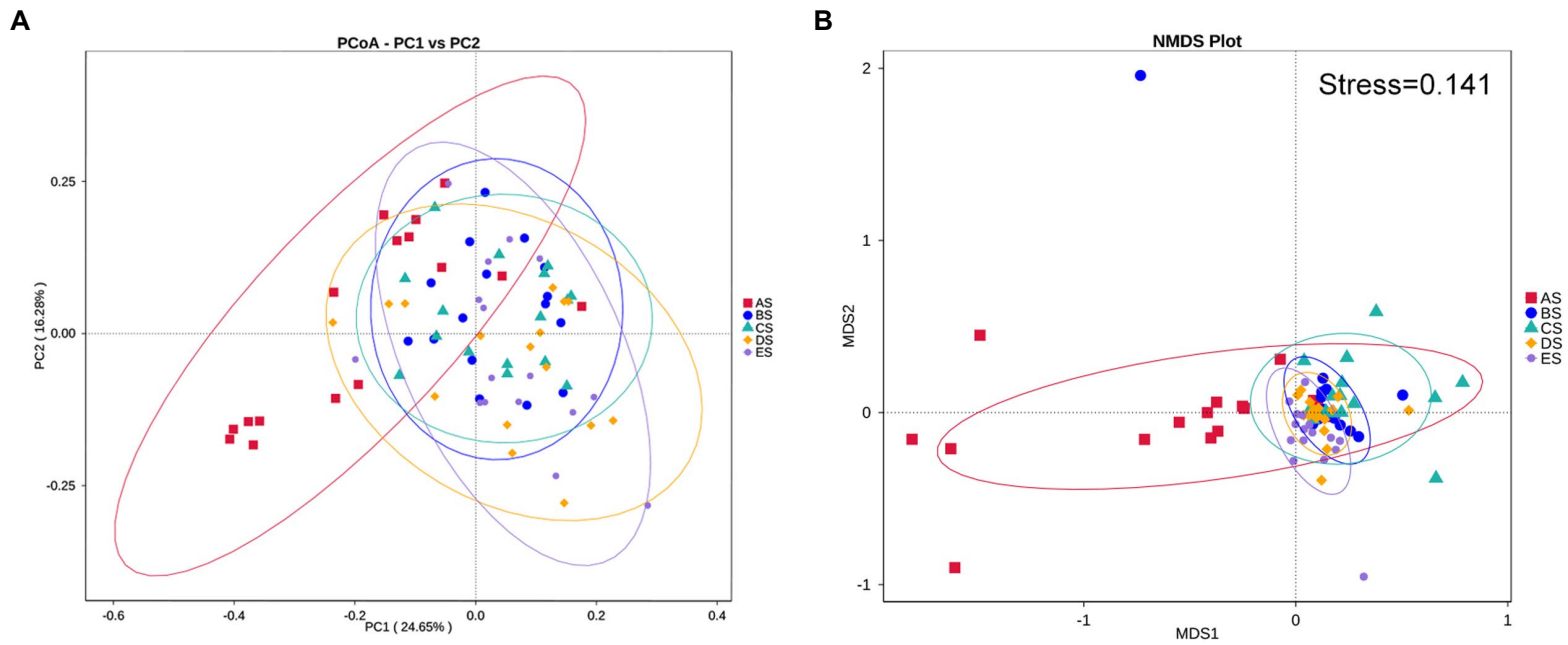
Structural discrepancy of microbial communities, assessed *via* by **(A)** PCoA based on weighted UniFrac distance and **(B)** NMDS based on Bray–Curtis distance. Each point represents one sample, and the same color represents the samples of the same group.

To assess the effect of microgravity on salivary bacterial diversity, we performed alpha and beta diversity analyses. Comparisons of alpha diversity among the five groups were presented by the Observed species, Chao1, Simpson, and PD whole-tree indices. Alpha diversity between any two groups did not differ significantly (*p* > 0.05, Figure 1). Weighted PCoA and NMDS represent beta diversity, that is, the difference in microbial community structure between the different groups. The distance between the groups in Figure 2 depicts the similarity of the bacterial community structure. Compared with the AS group, the community structures of groups BS, CS, DS and ES were significantly different. The community structures of BS, CS, DS, and ES were similar. This result was also confirmed by the dissimilarity analysis (Supplementary Table 2). At the same time, ANOSIM in Supplementary Table 2 also indicates that the difference between sample groups was greater than within each sample group, which could exclude the impact of individual differences in salivary microbiomes within group samples.

### Transition of the salivary microbiome

**Figure 3:**
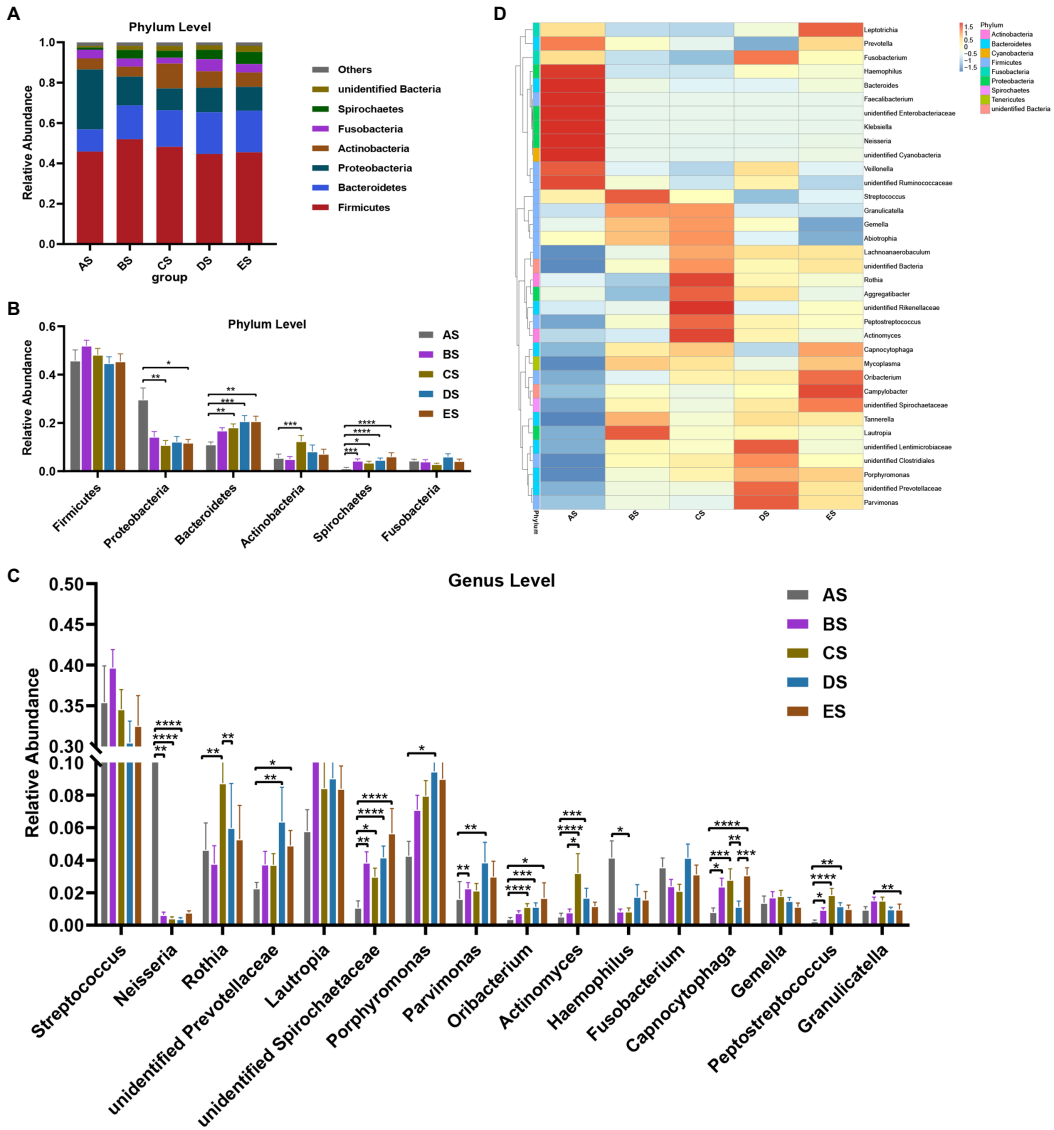
**(A)** Distribution of the dominant bacteria (mean relative abundance >1%) by the barplot at the phylum level. **(B)** Differential analysis of the dominant bacteria in saliva samples at the phylum level. **(C)** Distribution of the top 35 genera among the five groups, as depicted by the heatmap, at the genus level. **(D)** Differential analysis of the dominant bacteria in saliva samples at the genus level. Others represent the sum of relative abundance of all other genera except the seven dominant genera shown in the figure. Statistical analyses were performed using repeated-measures one-way ANOVA followed by Tukey’s post-hoc test or Friedman test with Dunn’s post-hoc test. *p < 0.05, **p < 0.01, ***p < 0.001, ****p < 0.0001.

The distribution and relative abundance of microbial taxa in each group at the phylum and genus levels were compared. First, the dominant microbiome was defined as the taxa with a mean relative abundance of >1% in all samples. The distribution of dominant bacteria at the phylum level is presented in Figure 3A, with Firmicutes accounting for the highest proportion (45.82% in AS, 51.95% in BS, 48.20% in CS, 44.69% in DS, and 45.45% in ES), followed by Bacteroidetes, Proteobacteria, Actinobacteria, Fusobacteria, and Spirochaetes. These bacteria accounted for 96.20% of the total sequences. As shown in Figure 3B, the relative abundance of Proteobacteria in the AS group was higher than that in the other groups. It was significantly lower in the CS and ES groups than in the AS group (*p* < 0.05). The relative abundance of Bacteroidetes progressively increased from the AS group to the ES group. It was significantly greater in the CS, DE, and ES groups than in the AS group (*p* < 0.05). The relative abundance of Actinobacteria in the CS group was significantly higher than that in the AS group (*p* < 0.05). The relative abundance of Spirochaetes in the AS group was significantly lower than that in the other groups (*p* < 0.05).

The distribution of the top 35 genera among the five groups is presented in a heat map (Figure 3C). *Streptococcus* and *Lautropia* were the two predominant genera in the salivary microbiome, with a mean relative abundance of >8% in all samples, followed by the subpredominant genera *Porphyromonas* and *Rothia* with a mean relative abundance of >5%. There were 16 dominant genera, and 12 of the 16 taxa exhibited decreases or increases in abundance after HDBR (Figure 3D). Compared with the AS group, the relative abundance of four genera (*unidentified Spirochaetaceae*, *Parvimonas*, *Capnocytophaga*, and *Peptostreptococcus*) increased significantly, and *Neisseria* decreased significantly in the BS group; six genera (*Rothia*, *unidentified Spirochaetaceae*, *Oribacterium*, *Actinomyces*, *Capnocytophaga*, and *Peptostreptococcus*) increased significantly, and two genera (*Neisseria* and *Haemophilus*) decreased significantly in the CS group, seven genera (*unidentified Prevotellaceae*, *unidentified Spirochaetaceae*, *Porphyromonas*, *Parvimonas*, *Oribacterium*, *Actinomyces*, and *Peptostreptococcus*) increased significantly, and *Neisseria* decreased significantly in the DS group; and four genera (*unidentified Prevotellaceae*, *unidentified Spirochaetaceae*, *Oribacterium*, and *Capnocytophaga*) increased significantly in the ES group. In addition, the relative abundance of *Actinomyces* in the CS group was significantly higher than that in the BS group, and the relative abundances of *Rothia* and *Capnocytophaga* were significantly higher than those in the DS group. The relative abundance of *Granulicatella* in the ES group was significantly lower than that in the BS group, and the relative abundance of *Capnocytophaga* was significantly higher than that in the DS group.

### Networks analysis

Network analysis provided details on the interactions among taxa by calculating the Spearman correlation coefficient. And the network diagrams (Supplementary Figures 1–5) provide a clearer picture of the association status of a genus with other genera. According to the statistics, there were 94 nodes and 533 edges in the AS group, 78 nodes and 157 edges in the BS group, 74 nodes and 125 edges in the CS group, 78 nodes and 131 edges in the DS group, and 73 nodes and 151 edges in the ES group. The line chart showed that the number of correlations between genera decreased gradually from the AS group to the CS group, and then recovered with time, but the number in the ES group was still lower than that in the AS group (Supplementary Figure 6). Co-occurrence relationships among the 16 dominant genera with a mean relative abundance >1% in all samples were further observed. The positive and negative correlations between the specific dominant genera are shown in Supplementary Table 3. It could also be concluded that the number of correlations between dominant genera was consistently reduced after HDBR and was still not fully recovered in the ES group. More importantly, the positive and negative relationships between the dominant genera changed dynamically with increasing bed rest time. For example, *Oribacterium* was positively correlated with *Actinomyces* and *Peptostreptococcus* before HDBR, and both became uncorrelated after HDBR. *Parvimonas* and *Neisseria* were negatively correlated before HDBR and were uncorrelated after HDBR. *Parvimonas* did not correlate with *Haemophilus* before HDBR but exhibited a significant negative correlation on day 6 of recovery after 15 days of simulated microgravity. This may indicate that the balance of the salivary microbial community is gradually disturbed and not restored in a short time.

### Changes in bacterial metabolism

**Figure 4:**
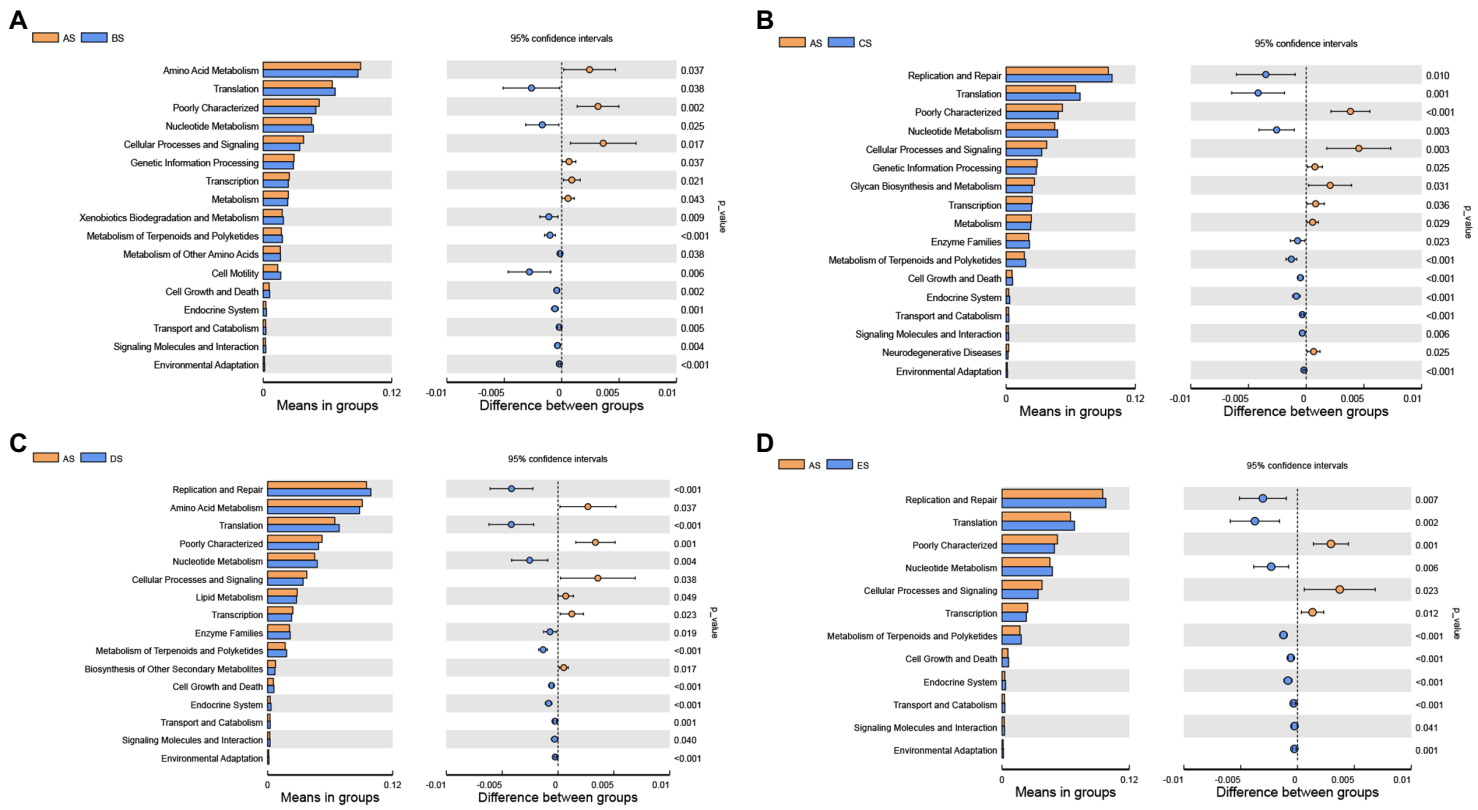
Differential analysis of the KEGG second-level functional annotations from the PICRUSt analysis. Differences in bacterial metabolism between the AS and BS groups **(A)**; AS and CS groups **(B)**; AS and DS groups **(C)**; and AS and ES groups **(D)**. Statistical analyses were performed using a paired Student’s *t*-test. *p* < 0.05 indicates significant differences.

PICRUSt analysis was performed to predict changes in the metabolic function of the salivary microbiome based on genetic information on OTUs in the Greengenes database. At the second level, differences in gene function were compared across groups (Figure 4). Membrane transport and carbohydrate metabolism, the top two gene functions in terms of mean relative abundance, were not significantly altered by HDBR. The relative abundance of translation and nucleotide metabolism gene functions was significantly lower in the AS group than that in the other groups (*p* < 0.05). In contrast, the relative abundance of poorly characterized and cellular processes and signaling gene functions was significantly higher in the AS group than that in the other groups (p < 0.05). Compared to the AS group, the relative abundance of replication and repair gene function was significantly increased in the CS, DS, and ES groups (p < 0.05), whereas the relative abundance of amino acid metabolism gene function was significantly decreased in the BS and DS groups (p < 0.05).

## Discussion

In this study, we described the changes in salivary secretion function and the salivary microbiome in young men under simulated microgravity and preliminarily explored the effects of these changes on oral health. Earlier studies by Dawes (1972) illustrated that human salivary flow rate and composition are affected by circadian rhythms. Daily fluctuations in the human salivary microbiome correlate with circadian rhythms (Sarkar et al., 2021). Therefore, the time for saliva collection was fixed in this study. Smoking, age, body size, diet, and oral hygiene affect the human salivary microbiome (Willis et al., 2018; Raju et al., 2019; Murugesan et al., 2020). Therefore, the inclusion criteria for the recruitment of participants and the confounding factors for the participants during the experiment were controlled accordingly.

The 15-day period of HDBR in this study had a negligible effect on the salivary secretion function of the individuals. Statistically, there were no differences in salivary pH or salivary flow rate between any two groups. However, there was a trend toward lower salivary pH and higher salivary flow rate on days 5, 10, and 15 of HDBR compared to the day before HDBR, and all returned to pre-levels on day 6 of recovery. A 56-day space mission monitored the oral health of three astronauts and observed a decrease in salivary flow rate during the flight (Brown et al., 1974). Huai et al. (2012) reported a significant reduction in salivary flow rate in individuals on the 3rd and 15th days of HDBR compared to pre-HDBR in a 30-day HDBR test, with later adaptive recovery. The variable salivary flow rate in these studies can be attributed to heterogeneity in study design, such as the time of saliva collection (pre-or post-meal), method of salivary secretion (rubber band chewing or no stimulation), age of the subject, time of exposure to microgravity, and environmental factors. Acidic pH causes enamel dissolution, and a reduction in pH plays a vital role in oral diseases, such as dental caries and periodontitis (Takahashi and Schachtele, 1990; Hajishengallis et al., 2017). In the present study, salivary pH decreased during HDBR but was still weakly alkaline. This may be ascribed to the relatively short time spent in the head-down position in the bed, where the salivary buffer system is less affected and still sufficient to regulate oral pH.

The present study showed no significant changes in the alpha diversity of the salivary microbiome. That is, the richness and diversity of the salivary microbial community remained stable after exposure to microgravity. Beta diversity analysis demonstrated that the salivary microbial community structure on days 5, 10, and 15 of HDBR differed significantly from that of pre-HDBR. A previous study of salivary microbial changes in 10 astronauts on 2–9-month missions showed a significant increase in alpha diversity and no difference in beta diversity during spaceflight (Urbaniak et al., 2020). The changes in saliva bacterial diversity observed in this study are quite different from those reported by Urbaniak et al., likely due to several external influencing factors. First, the saliva collection methods were different. The saliva collected in this study was non-stimulated whole saliva that flowed naturally. In contrast, the saliva collection in the space station was mainly obtained by placing cotton rolls in the mouth of astronauts until saturation and centrifuging them after returning to Earth. Furthermore, astronauts in the study by Urbaniak et al. were exposed to microgravity for extended periods. Lastly, the space environment is more complex than the simulated microgravity on the ground, as astronauts are exposed to microgravity, as well as radiation, noise, circadian rhythms, and other factors. Avila-Herrera et al. (2020) used shotgun metagenomic sequencing to study the salivary changes of an astronaut at eight time points before, during, and after a 135-day flight mission and detected that alpha diversity decreased during flight and gradually recovered after the flight but did not reach pre-flight levels during the observation period. However, this study was only conducted at the individual level, and more reproducible validation needs to be performed. Morrison et al. (2021) used shotgun metagenomic sequencing and microarray detection methods to investigate the salivary changes of four astronauts at eight time points before, during, and after the flight during a six-month mission and discovered that when saliva samples from the four astronauts were analyzed together, there was no change in alpha diversity; when the samples were analyzed individually, alpha diversity did not change consistently across the four astronauts but returned to pre-flight levels upon return to Earth, and beta diversity also changed due to flight status. In this study, the changes in salivary alpha and beta diversity were more consistent with those reported by Morrison et al.

Further analysis of the different species between groups revealed a dynamic and complex process of changes in dominant salivary bacterial abundance over the 28-day experiment. In this study, the relative abundance of *Rothia* tended to decrease on day 5 of HDBR compared to the day before HDBR. However, it increased significantly on day 10 of HDBR despite gradually decreasing to its original level over time. *Rothia*, a gram-positive bacterial genus from the phylum Actinomycetes, is generally more abundant in healthy periodontal subgingival microbe samples than in samples from individuals with periodontitis (Ikeda et al., 2020). However, they can also act as opportunistic pathogens and cause severe systemic infections in immunocompromised hosts (Ramanan et al., 2014). The reasons for the variation in the genus *Rothia* in this study are complex and require further exploration. At the same time, compared to the day before HDBR, the relative abundance of *Neisseria* decreased significantly with increasing bed rest time. The relative abundance of *Haemophilus* decreased significantly from pre-HDBR to day 10 of HDBR treatment. However, the abundance of all the above genera rebounded on day 6 of recovery. In a study by Morrison et al. (2021), when saliva samples from four astronauts were analyzed together, no significant changes in the relative abundance of taxa were observed between collection time points; in contrast, when analyzed individually, the saliva of two astronauts showed a decrease in the relative abundance of *Rothia*, *Neisseria*, and *Haemophilus* during the period spent on the international space station, which was also more similar to the results of the present study. *Neisseria* and *Haemophilus* are gram-negative bacteria from Proteobacteria, comprising aerobic and facultative anaerobic bacteria, respectively. Previous studies have reported higher relative abundances of *Neisseria* and *Haemophilus* in saliva samples from healthy individuals (Belstrøm et al., 2017; Shi et al., 2021). *Neisseria* has also been reported as a biomarker of healthy periodontal tissue (Meuric et al., 2017; Cai et al., 2021). Therefore, *Neisseria* and *Haemophilus* are beneficial bacteria, and their decrease may increase the incidence of oral diseases after exposure to microgravity.

In contrast, the relative abundance of the genus *Actinomyces* from the phylum Actinobacteria; the genera *Parvimonas*, *Peptostreptococcus*, and *Oribacterium* from the phylum Firmicutes; and the genera *Porphyromonas* and *Capnocytophaga* from the phylum Bacteroidetes all displayed a dynamic increase in their relative abundance during HDBR compared to pre-HDBR, but they varied. It is worth noting that the dominant genera that increased after HDBR were all anaerobic bacteria. *Capnocytophaga* is one of the resident microbes of the human oral cavity and is common in healthy individuals; some of its species may be associated with periodontal disease (Idate et al., 2020). Yang et al. (2021) recently reported that *Capnocytophaga* is significantly enriched in the saliva of patients with oral squamous cell carcinoma. In addition, the increased abundance of the genus *Oribacterium* has been associated with oral lesions in most oral microbial studies on disease (Hernandez et al., 2017; Yu et al., 2020). *Parvimonas micra* is the main species in the *genus Parvimonas* and is highly enriched in periodontitis and periapical lesions (Colombo et al., 2009; Rôças and Siqueira, 2018). Ko et al. (2020) showed that *Peptostreptococcus* was more abundant in periodontitis saliva, and another study considered it a biomarker of periodontitis after comprehensive analysis (Cai et al., 2021). Extensive studies have shown that *Porphyromonas gingivalis* and *Porphyromonas endodontalis*, from the genus *Porphyromonas, are* strongly correlated with the pathogenesis of periodontitis and apical periodontitis (Mysak et al., 2014; Bordagaray et al., 2021). Many species of the genus *Actinomyces* have a high affinity for the tooth surface and dominate root and dentin caries (Do et al., 2017; Liu et al., 2020). Overall, most of the increased bacterial counts after HDBR were associated with caries, apical periodontitis, and periodontitis, suggesting that microgravity may increase the risk of oral diseases. Nonetheless, most of these bacteria returned to pre-HDBR levels after the end of recovery, probably because of the short duration of HDBR. Additionally, many bacteria have not been identified at the genus level after HDBR. Since the specific mechanism of action of these bacteria is not precise, they have not been discussed in this paper; however, it cannot be assumed that these bacterial changes have no role in disease pathogenesis.

According to previous spaceflight studies and simulated microgravity experiments, there were few investigations and findings on the co-occurrence relationships between oral microbes and the prediction of microbial metabolic function. The interaction between bacteria is crucial for maintaining the ecological stability of the oral microbial community and can mediate the transition of the oral cavity from healthy to diseased ecological dysbiosis (Marsh and Zaura, 2017; Diaz and Valm, 2020). In the network analysis, numerous relationships were observed before HDBR, such as between *Oribacterium* and *Actinomyces* and between *Parvimonas* and *Neisseria*, suggesting that salivary microbiome in healthy individuals may require more complex interactions to maintain oral ecological homeostasis. After HDBR, the number of relationships between genera decreased and did not fully recover by day 6 of recovery. In addition, positive correlations between genera changed to negative or no correlations after HDBR, and new positive and negative correlations emerged for previously uncorrelated genera. Altered relationships between genera may also influence the composition and abundance of the salivary microbiome. Briefly, changes in these relationships may disrupt the original oral microbial community structure balance, lead to oral microecological dysbiosis, and increase the risk of oral diseases. In addition, functional predictions revealed that the relative abundance of most predicted gene functions was uniform across groups; in particular, the two most commonly identified gene functions did not change significantly in abundance after HDBR, indicating that the core functions of the salivary microbiome could remain stable under short-term simulated microgravity. However, after HDBR, gene functions, such as nucleotide metabolism, and translation, were significantly more abundant than pre-HDBR. The mechanism behind this phenomenon should be explored further in future studies.

In conclusion, complex changes in the salivary microbiome under HDBR conditions were observed in this study using 16S rRNA gene sequencing. The results indicate that the richness and diversity of salivary microbial communities remained stable in young men following 15 days in a simulated microgravity environment. In contrast, changes in the microbial community structure, microbial interactions, and predicted gene functions were observed. We acknowledge the limitations of this study. For example, the duration of HDBR in the current study was not long enough to produce longitudinal data for causal inference, thus making it impossible to determine whether changes in the salivary microbiome were directly associated with various diseases. In addition, many sequences obtained by 16S rRNA gene sequencing cannot currently be annotated at the species level. Consequently, in order to provide a theoretical basis for the prevention and treatment of oral diseases in astronauts, more HDBR tests must be carried out in the future. These tests must also use higher resolution microecological research methods to more thoroughly understand the changes in salivary bacterial communities under microgravity and to explore whether these changes could be a predictive factor of increased risk of caries or periodontitis at the individual level.

## Data availability statement

The datasets presented in this study can be found in online repositories. The names of the repository/repositories and accession number (s) can be found at: https://www.ncbi.nlm.nih.gov/, PRJNA879903.

## Ethics statement

The studies involving human participants were reviewed and approved by Space Science and Technology Institute (Shenzhen). The patients/participants provided their written informed consent to participate in this study. Written informed consent was obtained from the individual(s) for the publication of any potentially identifiable images or data included in this article.

## Author contributions

CT, PQ, and QZ contributed to the conception and design of the study. HS conducted the statistical analysis, interpreted the analysis results, and wrote the manuscript. DZ performed the statistical analysis. BX and BW organized the experiments. QZ performed the experiments. CT revised and reviewed the final manuscript. All authors contributed to the article and approved the submitted version.

## Conflict of interest

The authors declare that the research was conducted in the absence of any commercial or financial relationships that could be construed as a potential conflict of interest.

## Publisher’s note

All claims expressed in this article are solely those of the authors and do not necessarily represent those of their affiliated organizations, or those of the publisher, the editors and the reviewers. Any product that may be evaluated in this article, or claim that may be made by its manufacturer, is not guaranteed or endorsed by the publisher.

## Footnotes

^1^ http://www.arb-silva.de/
