# Supplementary material for: Short-term head-down bed rest microgravity simulation alters salivary microbiome in young healthy men
Source: Front Microbiol. 2022 Nov 10;13:1056637. doi: 10.3389/fmicb.2022.1056637 (PMC9684331; doi:10.3389/fmicb.2022.1056637)
Supplement: Supplementary file 1 [file Data_Sheet_1.docx]

Supplementary Material

# Supplementary Figures
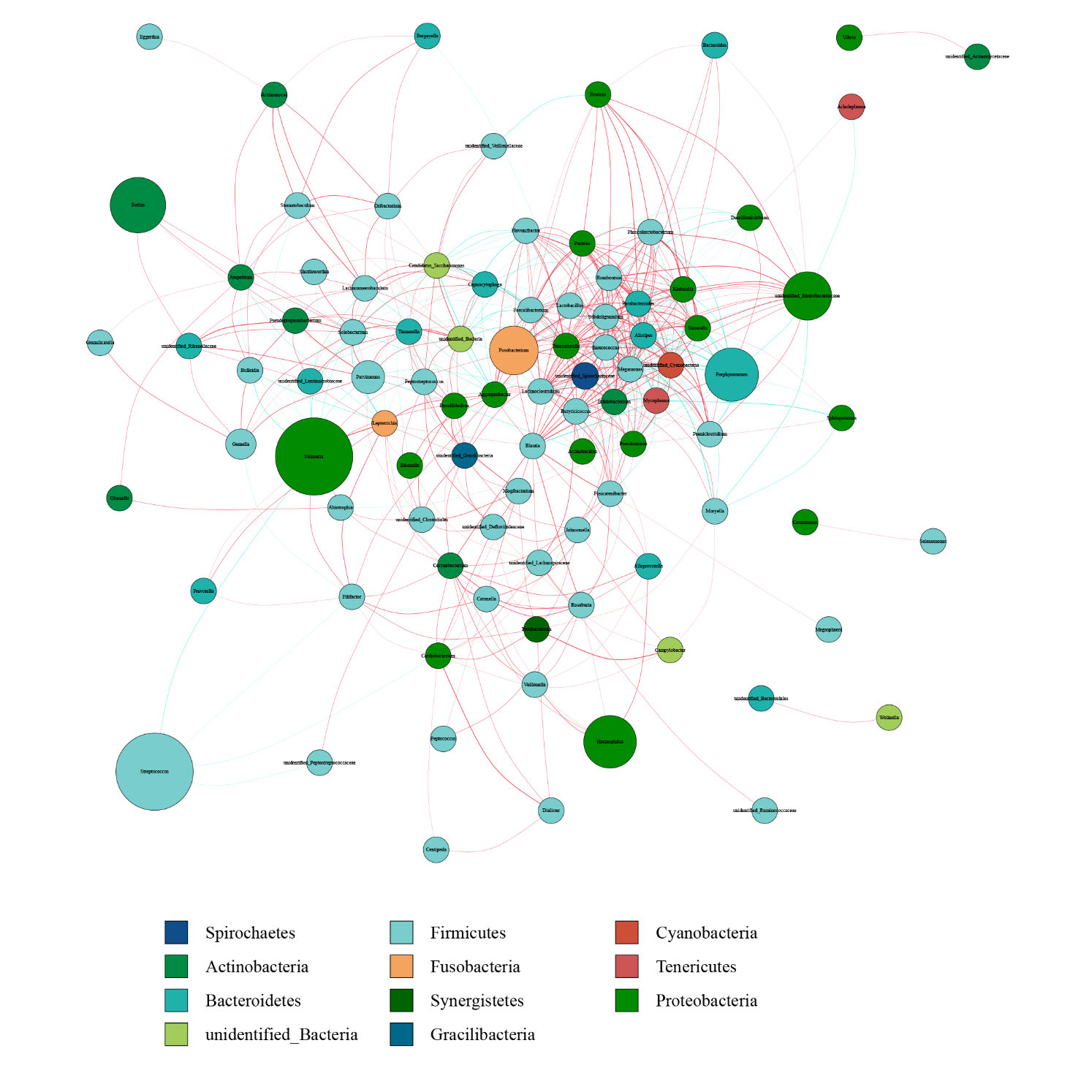


**Supplementary Figure 1.** Co-occurrence network of the AS group. Edges between each pair of genera indicate significant correlations (|R| > 0.6, P < 0.05). Each node represents a genus. The mean relative abundance determines the size of the nodes, and the nodes at the same phylum level have the same color. Edge thickness represents the absolute value of the correlation coefficient for species interactions, with red and blue lines indicating positive and negative correlations, respectively.

**
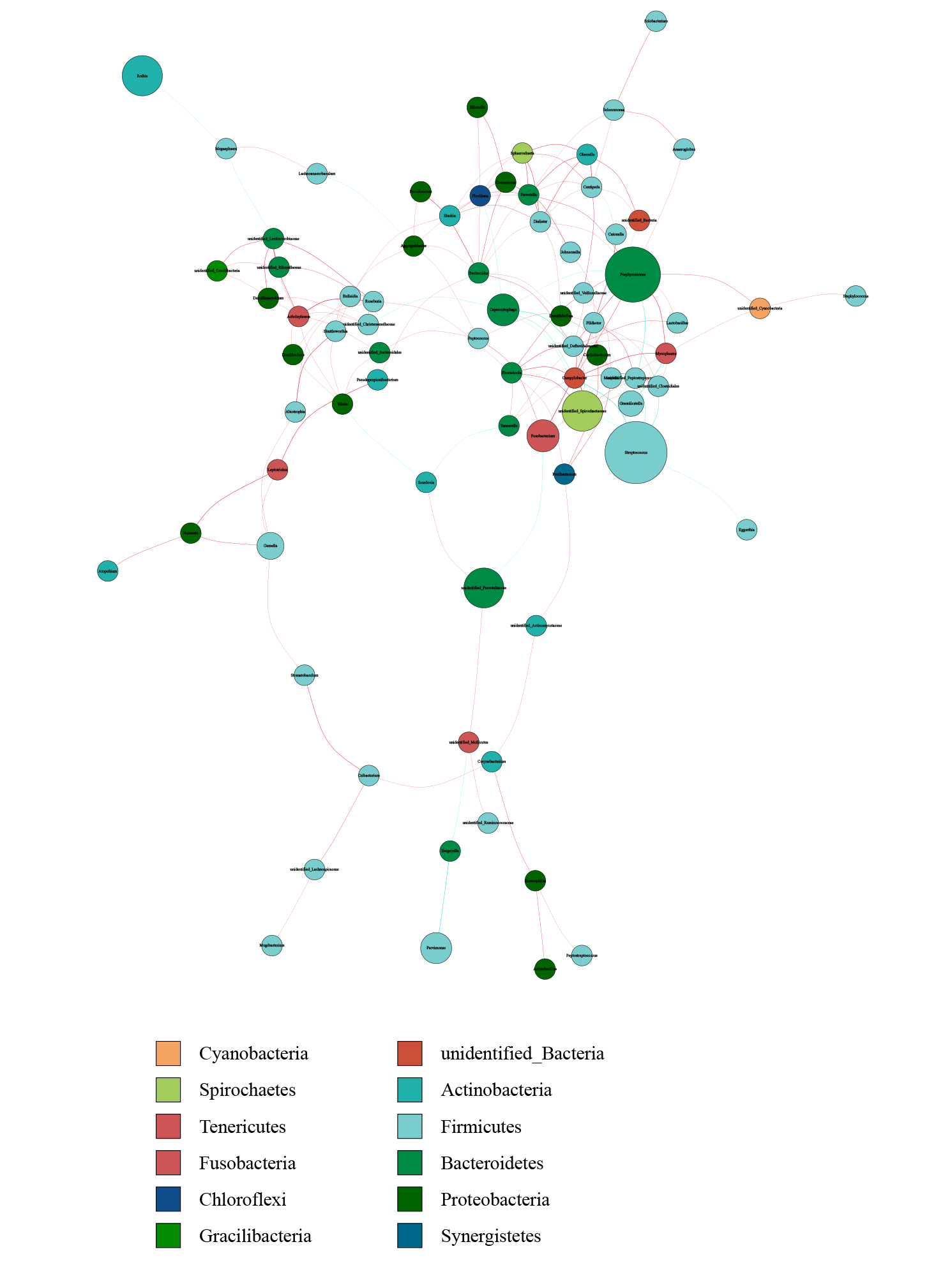
**

**Supplementary Figure 2.** Co-occurrence network of the BS group.


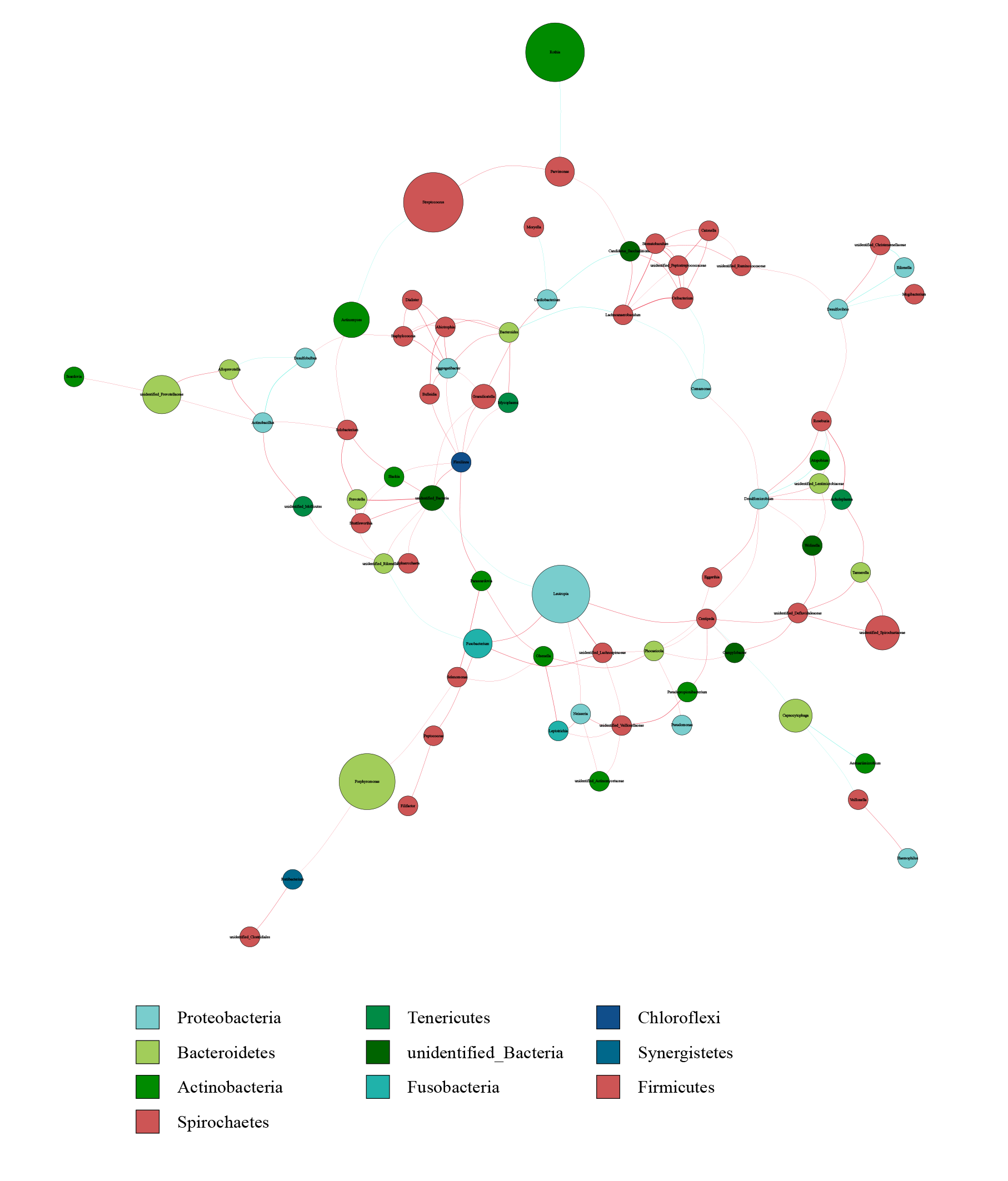


**Supplementary Figure 3.** Co-occurrence network of the CS group.


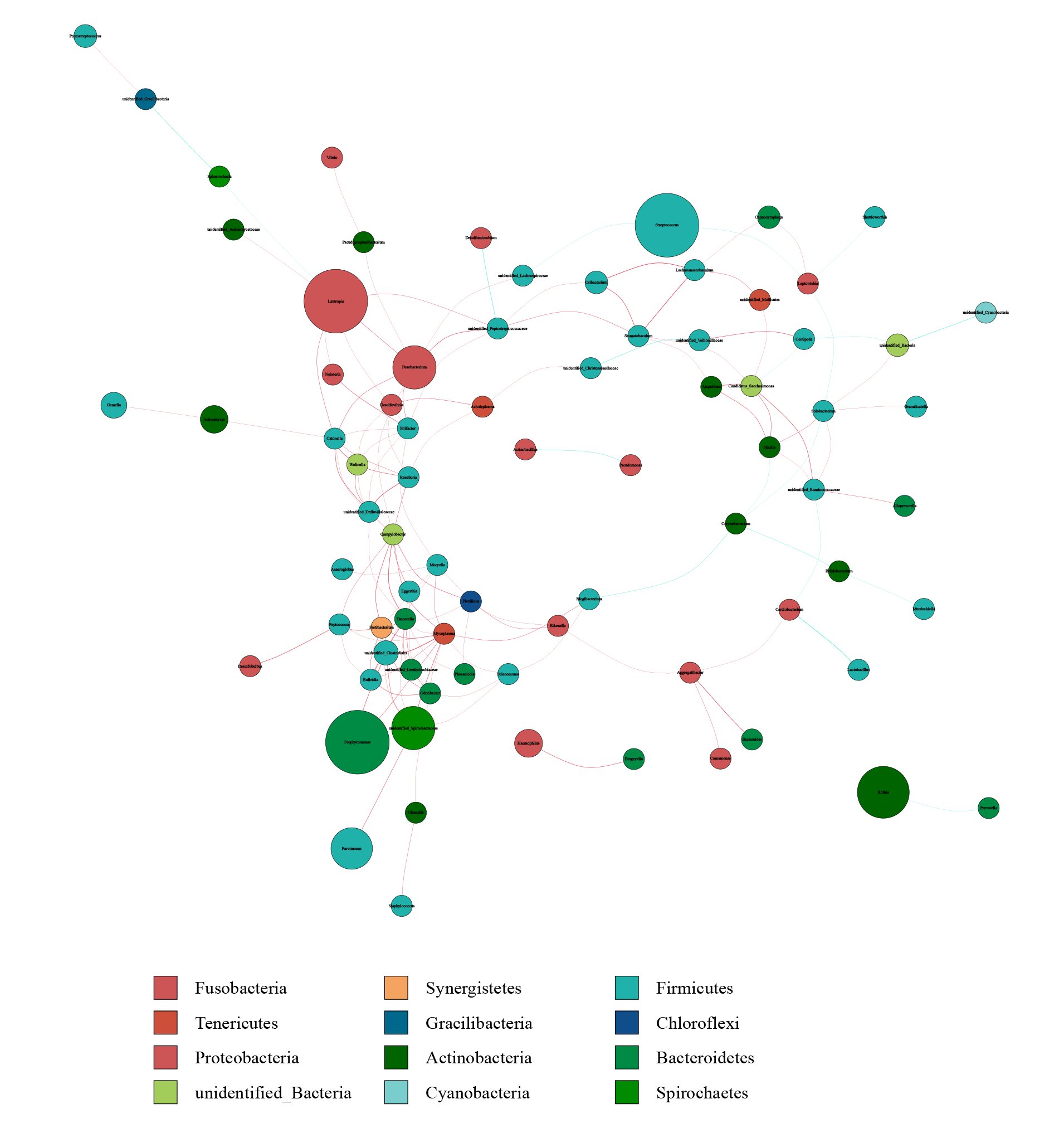


**Supplementary Figure 4.** Co-occurrence network of the DS group.


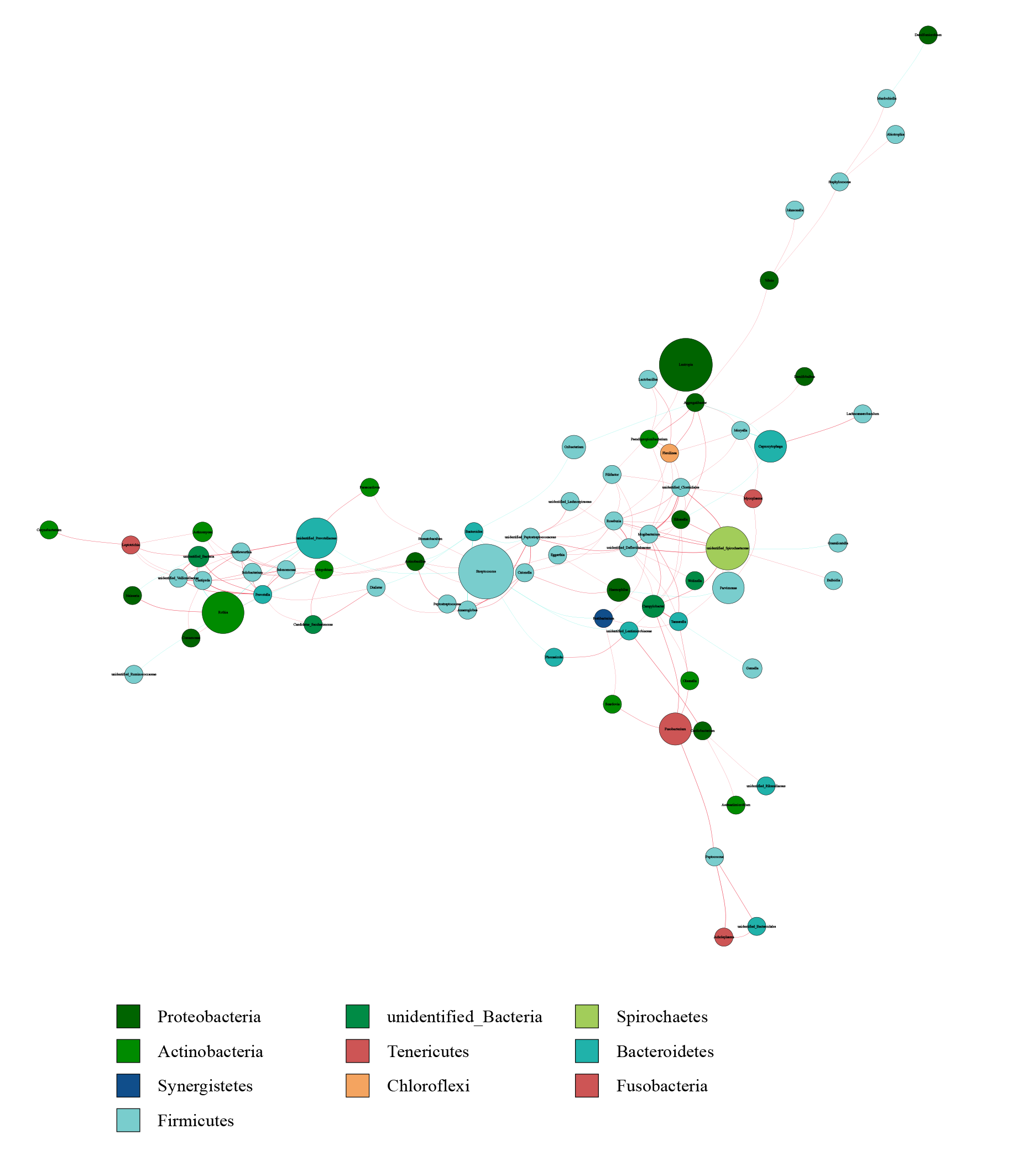


**Supplementary Figure 5.** Co-occurrence network of the ES group.


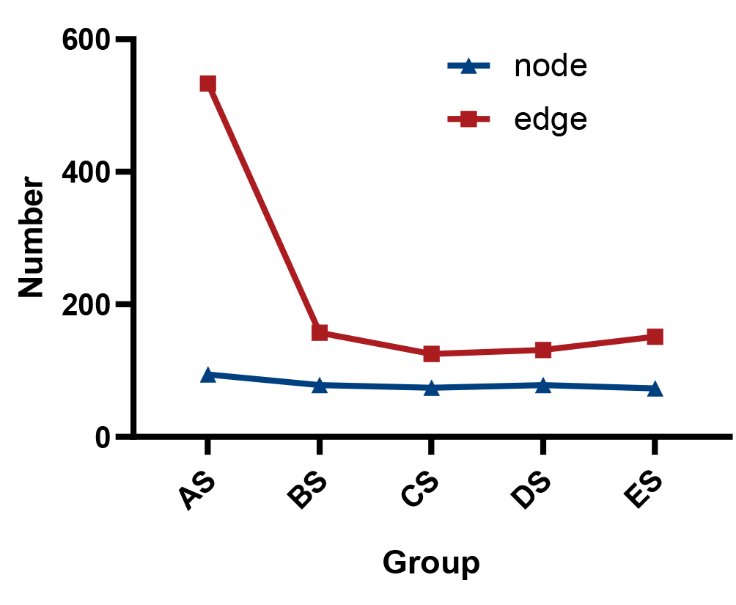


**Supplementary Figure 6.** The number of nodes and edges in the network diagram of the five groups.

**Supplementary Tables**

**Supplementary Table 1.** Data pre-processing statistics and quality control. Raw PE indicates the original downstream PE reads. Raw tags are the sequence of tags obtained by splicing. Clean tags are the sequence of raw tags after filtering low quality and short length. Effective tags are the sequence of tags finally used for subsequent analysis after filtering chimeras. Base is the number of bases in the final effective data. AvgLen is the average length of effective tags. Q20 and Q30 are the base quality values of effective tags greater than 20 (sequencing error rate less than 1 %) and 30 (sequencing error rate less than 1 %). GC (%) indicates the content of GC bases in effective tags. Effective (%) indicates the percentage of the number of effective tags to the number of raw PE.

**Supplementary Table 2.** Dissimilarity analysis of bacterial community structure between groups. The analysis of both ADONIS and ANOSIM is based on the Bray–Curtis distance. The R-value is between (-1, 1). R > 0 indicates significant differences between groups. R < 0 indicates that intra-group differences are greater than inter-group differences. R^2^ represents the degree of explanation of sample differences by different groups, that is, the ratio of group variance to total variance. P < 0.05 indicates significant differences.

**Supplementary Table 3.** Correlations between dominant genera (relative abundance > 1 %) in five groups. The R-value represents the Spearman correlation coefficient. |R| > 0.6 and P < 0.05 between the two dominant genera are shown in the table. |R| < 0.6 or P > 0.05 between the two dominant genera is represented by “-”.
